# Encoding of ultrasonic vocalizations in the auditory cortex

**DOI:** 10.1152/jn.00483.2012

**Published:** 2013-01-16

**Authors:** Isaac M. Carruthers, Ryan G. Natan, Maria N. Geffen

**Affiliations:** ^1^Department of Otorhinolaryngology and Head and Neck Surgery, University of Pennsylvania Perelman School of Medicine, Philadelphia, Pennsylvania;; ^2^Graduate Group in Physics, University of Pennsylvania, Philadelphia, Pennsylvania;; ^3^Graduate Group in Neuroscience, University of Pennsylvania Perelman School of Medicine, Philadelphia, Pennsylvania; and; ^4^Department of Neuroscience, University of Pennsylvania Perelman School of Medicine, Philadelphia, Pennsylvania

**Keywords:** auditory cortex, communication, modeling, ultrasonic vocalizations

## Abstract

One of the central tasks of the mammalian auditory system is to represent information about acoustic communicative signals, such as vocalizations. However, the neuronal computations underlying vocalization encoding in the central auditory system are poorly understood. To learn how the rat auditory cortex encodes information about conspecific vocalizations, we presented a library of natural and temporally transformed ultrasonic vocalizations (USVs) to awake rats while recording neural activity in the primary auditory cortex (A1) with chronically implanted multielectrode probes. Many neurons reliably and selectively responded to USVs. The response strength to USVs correlated strongly with the response strength to frequency-modulated (FM) sweeps and the FM rate tuning index, suggesting that related mechanisms generate responses to USVs as to FM sweeps. The response strength further correlated with the neuron's best frequency, with the strongest responses produced by neurons whose best frequency was in the ultrasonic frequency range. For responses of each neuron to each stimulus group, we fitted a novel predictive model: a reduced generalized linear-nonlinear model (GLNM) that takes the frequency modulation and single-tone amplitude as the only two input parameters. The GLNM accurately predicted neuronal responses to previously unheard USVs, and its prediction accuracy was higher than that of an analogous spectrogram-based linear-nonlinear model. The response strength of neurons and the model prediction accuracy were higher for original, rather than temporally transformed, vocalizations. These results indicate that A1 processes original USVs differentially than transformed USVs, indicating preference for temporal statistics of the original vocalizations.

adult rats communicate via ultrasonic vocalizations (USVs) (Knutson et al. 2002; Portfors 2007; Sewell 1970; Takahashi et al. 2010). Male rats emit high-frequency USVs during positive social, sexual, and emotional situations (Barfield et al. 1979; Bialy et al. 2000; Brudzynski and Pniak 2002; Burgdorf et al. 2000, 2008; Knutson et al. 1998, 2002; McIntosh et al. 1978; Parrott 1976; Sales 1972; Wohr et al. 2008). Despite their behavioral prevalence, little is known about the neural responses to USVs in the primary auditory cortex (A1) of the rat. While immediate-early gene expression has been shown to be elevated in A1 after exposure to USVs (Sadananda et al. 2008), the neural correlates of responses to USVs in rats have previously been identified only in the perirhinal cortex (Allen et al. 2007) and the amygdala (Parsana et al. 2012). Understanding how neurons in A1 encode vocalizations is essential for comprehending the function of areas that receive direct and indirect input from A1, and how perceptual correlates of vocalizations are formed in the downstream areas (Doupe and Kuhl 1999). Here, we characterize the responses of A1 neurons to rat USVs.

Neurons in A1 in other species exhibit strong responses to conspecific vocalizations (Gehr et al. 2000; Glass and Wollberg 1983; Huetz et al. 2009; Medvedev and Kanwal 2004; Pelleg-Toiba and Wollberg 1991; Wallace et al. 2005; Wang et al. 1995). However, the extent to which neural coding is specialized to encode conspecific vocalizations in A1 is a debated topic (Huetz et al. 2009; Wang et al. 1995). In mice, ultrasonic pup calls elicit strong responses in neurons in A1 of adult female mice (Galindo-Leon et al. 2009; Liu and Schreiner 2007; Liu et al. 2006). While rat USVs also exhibit a large amount of diversity in their spectro-temporal structure (Wright et al. 2010), how those spectro-temporal fluctuations relate to A1 responses is not understood. The parameters of rat USVs can be characterized with high precision, and in the present study they serve as stimuli that permit direct evaluation of the temporal dynamics in neuronal activity in A1.

We recorded neuronal activity in response to USVs in their original and transformed configurations from the awake rat A1. The transformed stimuli maintained the same first-order statistics as the original vocalizations but differed in either direction (original vs. reverse playback) or temporal modulation (playback speed). A large fraction of A1 neurons were responsive to and selective for a subset of USVs. Their responses were accurately predicted by a feedforward model based on the dominant frequency modulation and amplitude of the sound and were more accurately predicted for responses to the original or reversed, rather than temporally transformed, versions of the USVs.

## METHODS

### Animals

All procedures were approved by the Institutional Animal Care and Use Committee of the University of Pennsylvania. Subjects in all experiments were adult male rats. Rats were housed in a temperature- and humidity-controlled vivarium on a reversed 24-h light-dark cycle with food and water provided ad libitum.

### Surgery

**Fig. 1.**
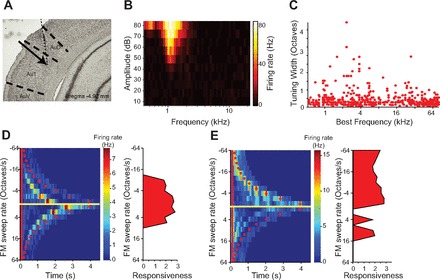
Recording location and tuning of recorded units. *A*: trace of the tetrode in a Nissl-stained, fixed coronal slice of the brain. The tetrode terminated in layer 5 of A1 (Au1, primary auditory cortex) (arrow). *B*: tuning curve of a representative unit. The color represents the response strength to a tone pip at a specific frequency (*x*-axis) and loudness (*y*-axis). *C*: distribution of the best frequency and tuning bandwidth of all recorded units that had significant response to the tuning curve stimulus. *D*: responses of a representative, frequency-modulated (FM) sweep rate-tuned unit to FM sweeps. *Left*: time course of the firing rate of the unit in response to FM sweeps at different rates. Red line indicates stimulus onset, and red dots indicate stimulus offset. *Right*: response strength of the unit to FM sweeps at different rates. *E*: responses of a representative FM direction-tuned unit to FM sweeps. Axes same as *D*.

Sprague-Dawley or Long-Evans adult male rats (*N* = 6, 12–16 wk) were anesthetized with an intraperitoneal injection of a mixture of ketamine (60 mg/kg body wt) and dexmedetomidine (0.25 mg/kg). Buprenorphine (0.1 mg/kg) was administered as an operative analgesic, with ketoprofen (5 mg/kg) as postoperative analgesic. Rats were implanted with chronic custom-built multitetrode microdrives as previously described (Otazu et al. 2009). The animal's head was secured in a stereotactic frame (David Kopf Instruments). After the recession of the temporal muscle, craniotomy and durotomy were performed over the location of A1. A microdrive housed eight tetrodes, of which two were used for reference and six for signal channels. Each tetrode consisted of four polyimide-coated nichrome wires (Kanthal Palm Coast, wire diameter of 12 μm) twisted together and was controlled independently with a turn of a screw. Two screws (one reference and one ground) were inserted in the skull at locations distal from A1. The tetrodes were positioned 4.0–6.5 mm posterior to bregma and 6.0 mm left of the midline and covered with agar solution (3.5%), and the microdrive was secured to the skull with dental acrylic (Metabond) and dental cement. The location of the electrodes was verified on the basis of stereotaxic coordinates, the electrode position in relation to brain surface blood vessels, and through histological reconstruction of the electrode tracks (Fig. 1*A*). During the recording, the microdrive was connected via a custom-built interface board to a headstage (Neuralynx). The electrodes were gradually advanced below the brain surface in daily increments of 40–50 μm. The location was also confirmed by identifying the frequency-tuning curve of the recorded units (Fig. 1*B*). The recorded units' best frequency (frequency of the tone that elicited the highest firing rate; see below) and tuning width spanned the range of rat hearing (Fig. 1*C*) and was consistent with previous studies (Sally and Kelly 1988).

### Stimulus Construction

#### Original vocalizations.

The original vocalizations were extracted from a recording provided by Diego A. Laplagne (Rockefeller University). The recordings were collected when two adult male rats, housed in isolation, were placed in a single cage together for 2 h. Vocalizations were recorded with a free-field ultrasonic microphone (Avisoft Bioacoustics, CM15, sensitivity 50 mV/Pa, frequency range: 10–200 kHz, input-referred self-noise level 18 dB).

From the continuous recording, vocalizations were extracted for further analysis, separately for each rat. The recorded sound wave was transformed into a spectrogram with the multitapered spectrogram transform (Chronux toolbox; Bokil et al. 2010); the entropy of the signal across all spectral channels was computed and subjected to a threshold. The onset of the vocalizations was taken as the time at which the threshold was reached. The threshold was manually adjusted to capture all vocalizations that were visually observed as distinct in the spectrogram of the signal, after which the analysis was fully automatic. The minimum intervocalization separation for detection was set to 40 ms, so the onset of each vocalization was identified by a threshold crossing that was at least 40 ms after the previous time the spectral entropy exceeded threshold. For initial response characterizations, 8 vocalizations were isolated at random from the long recording; 350 additional vocalizations were isolated for subsequent response characterization.

**Fig. 2.**
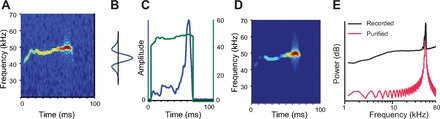
Construction of noiseless vocalizations. *A*: spectrogram of a representative recorded vocalization. *B*: spectral filter used to determine the instantaneous frequency and its amplitude of the vocalization in each 1-ms time bin. *C*: time course of maximum frequency and amplitude used to construct the noiseless vocalization. *D*: spectrogram of the noiseless vocalization version. *E*: power spectrum of the recorded and noiseless (purified) vocalization.

A noiseless version of the vocalizations was constructed to ensure that the neural responses were due to vocalizations and not due to an interaction with background noise (Bar-Yosef and Nelken 2007). To generate the noiseless stimuli, using an automated procedure, we isolated the dominant frequency and amplitude for each noisy vocalization (Fig. 2). The noiseless signal was constructed as a frequency- and amplitude-modulated tone, such that at any time, the frequency, *f*(*t*), and amplitude, *a*(*t*), of that tone were matched to the peak amplitude and frequency of the recorded USV. In each 1.0-ms bin, the values of *f*(*t*) and *a*(*t*) were extracted from a multitapered spectrogram of the vocalizations, generated with a 2.0-ms window in steps of 0.25 ms, by convolving each temporal slice of the spectrogram with a ridge-detecting filter (Fig. 2*B*). They were then resampled back up to our system playback frequency (400 kHz) with a shape-preserving piecewise cubic interpolation (Fig. 2*C*). The noiseless signal was generated as *x*(*t*) = *a*(*t*)sin[2π∫_0_^*t*^*f*(τ)dτ] (Fig. 2*D*). The power spectrum of the noiseless vocalization matched that recorded in the USV range, whereas the noise power sidebands were removed (Fig. 2*E*). Eight noiseless vocalizations were constructed from the originally recoded vocalizations (see Fig. 4*A*).

#### Long vocalization sequence.

**Fig. 3.**
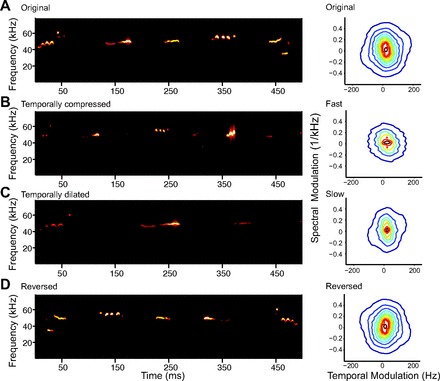
Spectro-temporal content of original and transformed vocalizations. *A*, *left*: spectrogram of a sequence of 4 original vocalizations of the 350 vocalization sequence. Each vocalization is represented as a continuous amplitude and FM tone. *Right*: temporal and frequency modulation spectrum of the 350 vocalizations. *B*: spectrogram and modulation spectrum of temporally compressed (accelerated, ×1.5) vocalizations. *C*: spectrogram and modulation spectrum of temporally dilated (slowed down, ×0.67) vocalizations. *D*: spectrogram and modulation spectrum of reversed vocalizations.

Three hundred and fifty vocalizations were extracted from the original vocalization recording, and noiseless versions of these vocalizations were constructed as described above. Next, the vocalizations were concatenated into a long string with 50-ms intervocalization separation (Fig. 3*A*; see Fig. 6*A*, Fig. 8*A*). The 50-ms intervocalization separation was chosen to match the mean natural vocalization rate of 10 Hz (D. A. Laplagne, unpublished observations). The temporally dilated vocalizations (Fig. 3*C*) were generated as *x*(*t*) = *a*(0.67*t*)sin[2π∫_0_^0.67*t*^*f*(τ)dτ], and temporally compressed vocalizations (Fig. 3*B*) were generated as *x*(*t*) = *a*(1.5*t*)sin[2π∫_0_^1.5*t*^*f*(τ)dτ]. To generate the reverse vocalization sequence, the original calls were reversed in time, *x*(*T* − *t*) = *a*(*t*)sin[2π∫_0_^*t*^*f*(τ)dτ], and concatenated in the opposite order to form a sequence (Fig. 3*D*). The temporal and spectral modulation power spectrum was computed as the Fourier transform of the autocorrelation matrix of the spectrogram of the full stimulus (Singh and Theunissen 2003). The range of the temporal and frequency modulations of the stimuli differed for temporally compressed and dilated stimuli compared with the original and reversed (Fig. 3, *right*).

### Neural Recordings

Neural signals were acquired daily from 24 chronically implanted electrodes in awake, freely moving rodents with a Neuralynx Cheetah system. The neuronal signal was filtered between 0.6 kHz and 6.0 kHz, digitized, and recorded at 32-kHz rate. Spikes were clustered into single-unit and multiunit clusters with either Neuralynx Spike Sort 3D or Plexon Offline Spike Sorter software. We used a stringent set of criteria to isolate single units from multiunit clusters (Bizley et al. 2010; Brasselet et al. 2012; Otazu et al. 2009). Single-unit clusters contained <0.1% of spikes within a 1.0-ms interspike interval, and the spike waveforms had to form a visually identifiable distinct cluster in a projection onto a three-dimensional subspace.

The acoustical stimulus was delivered via a magnetic speaker (MF-1, Tucker-Davis Technologies) positioned above the recording chamber. The speaker output was calibrated with Bruel and Kjaer 1/4-in. free-field microphone type 4939, which was placed at the location that would normally be occupied by the animal's ear, by presenting a recording the speaker output of repeated white noise bursts and tone pips between 400 and 80,000 Hz. From these measurements, the speaker transfer function and its inverse were computed. The input to the microphone was adjusted using the inverse of the transfer function such that the speaker output 70-dB tones within 3 dB between 400 and 80,000 Hz. Spectral and temporal distortion products were measured in response to tone pips between 1 and 80 kHz and were found to be >50 dB below the SPL of the fundamental. All stimuli were presented at 400-kHz sampling rate, with custom-built software based on a commercially available data acquisition toolbox (MathWorks) and a high-speed data acquisition card (National Instruments).

### Quantification of Neural Response Strength

To compute the strength of neuronal responses to the individual USVs, the responses of neurons to 50–200 repeats of the eight USVs were recorded and binned in 10-ms bins. The baseline was taken 0.5–1.0 s after the vocalization onset. Each response was represented as a vector consisting of spike counts in 10-ms bins between 10 and 120 ms after stimulus onset. The minimum firing rate was set to 0.1 Hz during the response and at baseline. The response strength was calculated as the Mahalanobis distance between the response and the baseline. The Mahalanobis distance D_M_ of a vector $\vec{x}$ assumed to be from a distribution with mean $\vec{\mu }$ and covariance matrix S is computed as ${\text{D}}_{\text{M}}\left(\vec{x}\right)=\sqrt{{\left(\vec{x}-\vec{\mu }\right)}^{\text{T}}{\text{S}}^{-1}\left(\vec{x}-\vec{\mu }\right)}$. Responses were considered significant if this measure, normalized by the square root of the stimulus repeat number, exceeded 3. The response-eliciting USV fraction, *R*_USV_, was computed for each neuron as the number of USVs eliciting significant responses divided by the number of USVs presented. The response selectivity index, *RS*, was computed as the maximum response strength to a USV divided by the mean response strength to all USVs presented.

### Tone Pip Response Measurement

The firing rate of neurons was recorded in response to randomly interleaved 50-ms-long tone pips, with 250-ms intertone interval. For a subset of neurons (*N* = 147), amplitude- and frequency-tuning curves were collected: tone pips at 100 frequencies spaced uniformly in log-frequency space between 0.4 and 80 kHz were presented at 10 sound pressure levels each, uniformly distributed between 10 and 80 dB (relative to reference pressure of 20 μPa). The best frequency was computed as the frequency of the tone that evoked the maximum response strength averaged over SPL of 40 and 80 dB (Fig. 1*B*). For another subset of units (*N* = 424), tone pips were presented at a single sound pressure level of 50 dB (relative to reference pressure of 20 μPa) with 100 frequencies spaced uniformly in log-frequency space between 0.4 and 80 kHz. The response strength, which combined onset and offset responses, was computed as the mean firing rate of neurons during 0 to 80 ms after tone onset. The best frequency was computed as the frequency of the tone that evoked the maximum response strength (Brown and Harrison 2009). The tuning bandwidth was computed at 10% of the maximum of the peak, fitted to a Gaussian. The peak was considered significant if the maximum firing rate exceeded by 3 standard deviations the mean firing rate in response to frequencies outside the peak. The overlap between the spectral response profile and the power spectrum of vocalizations was computed as the dot product of the power spectrum of the USV waveform and the response strength of the neuron at each frequency (extrapolated to the frequencies of the USV power spectrum), normalized by the sum of the power spectrum of the USV across all frequencies.

### Frequency-Modulated Sweep Responses

The spiking responses of neurons were recorded in response to randomly interleaved frequency-modulated (FM) sweeps. The sweeps were presented at 500-ms intervals between the end and the beginning of successive sweeps. The sweeps were composed as a tone whose frequency was swept logarithmically between 1 kHz and 80 kHz. The sweep was padded at each end with 100-ms pure tone at the start or end frequency (1 kHz or 80 kHz, depending on the sweep direction). The sweeps were presented at 22 rates, log-uniformly distributed between +64 and −64 octaves/s. The firing rate for each FM sweep rate was computed by binning the spikes in 10-ms bins and smoothing them with a 3-bin Gaussian envelope. The response strength to each FM sweep rate, *R*_*i*_, was computed as the normalized mean baseline-subtracted firing rate in bins during the sweep presentation, during which the firing rate exceeded the 95% confidence limit of the baseline firing rate. The firing rate was normalized by the standard deviation of the baseline firing rate to facilitate comparison across units. A small offset term was added to the denominator to prevent division by 0. The 95% confidence limit was computed over the baseline firing rate, assuming that the baseline fluctuated as a Gaussian with the standard deviation computed over all trials. The FM rate tuning index, *I*_FM_, was computed over *n* sweep speeds in which the firing rate exceeded the 95% confidence limit (Atencio et al. 2007):

$$I_{\text{FM}\;\text{tuning}}=\frac{n}{n-1}\left(1-\frac{\langle R_{i}\rangle }{\max\left(R_{i}\right)}\right)$$

Brackets denote the average over all sweep rates.

The FM directionality index, *I*_D_, was computed over responses to sweeps, where *R*_*i*_^+^ denotes the response strength to an up sweep at rate *i* and *R*_*i*_^−^ denotes the response strength to a down sweep at rate *i* (Atencio et al. 2007; Nelken and Versnel 2000; Shamma et al. 1993):

$$I_{\text{D}}=\frac{\langle R_{i}^{+}\rangle -\langle R_{i}^{-}\rangle }{\langle R_{i}^{+}\rangle +\langle R_{i}^{-}\rangle }$$

Brackets denote the average over all up or down sweep rates.

### Linear-Nonlinear Models

#### Reduced-parameter generalized linear-nonlinear model.

A generalized linear-nonlinear model (GLNM) provides an ideal framework for constructing a predictive model of neuronal responses to the stimulus (Calabrese et al. 2011; Pillow et al. 2008, 2011), because of the non-Gaussian statistics of the stimulus. The output of the model is the activity of an individual neuron, while the input to the model is the stimulus, as represented by its envelope *a*(*t*) and differential frequency ω′(*t*) (see Fig. 8*B*). In the model each of two input parameters is convolved with a linear kernel, after which the outputs of the filters are summed (see Fig. 8*C*). This sum then undergoes a rectifying nonlinearity to approximate the spiking transformation (see Fig. 8*D*). The output of this function is entered into a Poisson generator to predict individual neuronal firing. The model prediction is calculated as:

$${\hat{r}}_{i}\left(t\right)=P\left(f\left(b+\int_{\tau =0}^{\tau }a\left(t-\tau \right)k_{i}^{a}\left(\tau \right)+\omega \prime \left(t-\tau \right)k_{i}^{\omega }\left(\tau \right)\text{d}\tau \right)\right)$$

where *r̂*_*i*_(*t*) is the response of neuron *i* at time *t*, *k*_*i*_ is the linear kernel of the neuron with respect to the stimulus, *T* is the length of the kernels, *b* is the baseline log-firing rate, *f*(*x*) is the instantaneous nonlinear function (here taken as an exponential), and *P* is a Poisson generator.

To fit GLNM, we used the maximum likelihood optimization approach (Pillow et al. 2008). With this approach, the model parameters are determined such that they maximize the likelihood of the recorded spike trains given the prediction of the model. We approximated the log-likelihood of the spike train as *L* = ∑*_t_i__* ln*r̂_i_*(*t_i_*) − ∫*r̂_i_*(*t*)d*t*, where *t*_*i*_ are the spike times. We then calculated the gradient of the log-likelihood with respect to the model parameters and used standard iterative optimization algorithms to find the optimal model, maximizing the likelihood of the spiking response over 100 trials given the model's prediction. To be included in the analysis, a unit had to have a mean discharge rate of at least 0.1 Hz during the stimulus. At each iteration the algorithm computed the log-likelihood and the gradient of the log-likelihood with respect to the model parameters and incremented the model's parameters along the steepest gradient. Increments were determined with the built-in MATLAB optimization procedure *fminunc* (MathWorks).

When maximizing *L* above, we also included an L2 regularization term: −γ∫_0_^*T*^*k*_*i*_^*a*^(τ)^2^ + *k*{_*i*_^ω^}(τ)^2^dτ, which served to reduce overfitting noise in the model. Because of computational constraints, the regularization coefficient γ was determined empirically by selecting the value that resulted in the model with the highest predictive accuracy in the case of a few exemplar cells. The accuracy of prediction of the model was computed as the coefficient of correlation between the model prediction and recorded responses to a novel stimulus.

For each neuron, spikes were binned in 1-ms bins and smoothed with a Gaussian window of 1-ms width. Because recent studies indicate that neurons in A1 carry information at <2 ms precision (Kayser et al. 2010), the smallest bin size (1 ms) was used for the analysis. Firing rate was computed for each trial and as an average across trials. The response strength to the USVs, *R*_*i*_, was computed as the normalized mean baseline-subtracted firing rate in bins during the USV presentation, during which the firing rate exceeded the 95% confidence limit of the baseline firing rate. The firing rate was normalized by the standard deviation of the baseline firing rate to facilitate comparison across units. A small offset term was added to the denominator to prevent division by 0. The 95% confidence limit was computed over the baseline firing rate, assuming that the baseline fluctuated as a Gaussian with the standard deviation computed over all trials. The significance of the response of the neuron was assayed by the signal-to-noise ratio, defined as the ratio of the standard deviation of the firing rate averaged over the vocalization sequence (corresponding to the square root of the power of the signal) divided by the standard error of the mean firing rate across trials (noise). A neuron was considered to respond significantly if the signal-to-noise ratio was higher than 2. The model was fitted on 200 vocalizations, and the predictive accuracy was computed as the mean over the remaining 150 vocalizations.

#### Spectrogram-based linear-nonlinear model.

To analyze the improvement in the model fit due to the low-dimensional parameterization of the stimulus, we also fitted a linear-nonlinear model (LNM) computed with standard reverse correlation technique (Baccus and Meister 2002; Escabi and Read 2003; Geffen et al. 2007; Theunissen et al. 2001), using a spectrogram as an input (see Fig. 8*E*). The filter was computed by normalizing the convolution of the response and the stimulus by the stimulus autocorrelation matrix (see Fig. 8*F*), and the instantaneous nonlinearity (see Fig. 8*G*) was computed directly from the firing rate vs. linear prediction plot (Baccus and Meister 2002; Geffen et al. 2007).

### Statistical Tests

The correlation coefficient (*r*) was computed as Pearson's correlation coefficient with standard MATLAB routines. Student *t*-test and multivariate analysis of variance (MANOVA) were conducted on either paired or unpaired samples (as indicated in text) with standard MATLAB routines. Repeated-measure analysis of variance (ANOVA) was performed in SPSS Statistics (IBM). Bonferroni multiple-comparison correction was used whenever appropriate.

## RESULTS

We measured and analyzed the responses of neurons in A1 to USVs emitted by male rats in a social context. We found that A1 neurons exhibited significant responses to USVs, typically selective for a subset of USVs. The temporal dynamics of A1 responses to a long sequence of USVs presented at the ethological rate were accurately predicted by an integrative model that took the amplitude and frequency modulation as the input (GLNM, see methods). The response strength, as well as the prediction accuracy of the model for each neuron, correlated with its FM rate tuning index and best frequency. A1 neurons' response strength and the model prediction accuracy were highest for the original, compared with temporally transformed, USVs, indicating a preference for the ethologically relevant parameters of USVs in the neuronal circuitry that underlies A1 responses.

### A1 Neurons Exhibit Reliable, Selective Responses to Ultrasonic Vocalizations

**Fig. 4.**
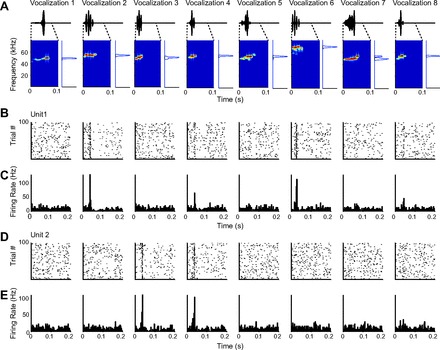
Responses of 2 representative neurons to 8 vocalizations. *A*: each subplot shows waveform of vocalizations (*top*), spectrogram of the first 100 ms of each vocalization (*bottom left*), and normalized power spectrum of each vocalization (*bottom right*). *B*: raster plots of responses of *unit 1. C*: peristimulus time histogram (PSTH) of responses of *unit 1*, binned in 3-ms time bins. *D*: raster plots of responses of *unit 2*, same as *B. E*: PSTH of responses of *unit 2*, same as *C*.

**Fig. 5.**
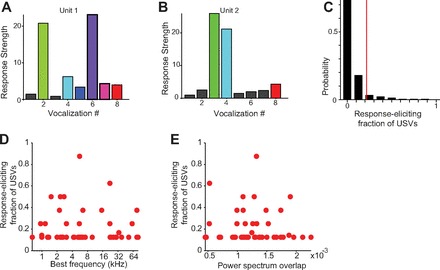
Response strength of recorded units to the vocalizations. *A*: response strength of *unit 1* to the vocalizations. *B*: response strength of *unit 2* to the vocalizations. *C*: histogram of the response-eliciting fraction of ultrasonic vocalizations (USVs) (*R*_USV_) for each recorded unit. Red bar, mean of the vocalization selectivity. *D*: *R*_USV_ for each unit vs. its best frequency. *E*: *R*_USV_ of each unit vs. the normalized overlap between its spectral response profile and the power spectrum of USVs.

Little is known about how rat USVs are encoded in A1. To assay whether A1 neurons responded to the USVs, we first presented eight vocalizations drawn at random from recordings (Fig. 4*A*) to awake, freely moving rats and recorded neuronal responses in A1, using the chronically implanted multitetrode microdrives. The units were localized to A1 (Fig. 1*A*), exhibited frequency-tuning curves typical of A1 neurons (Fig. 1*B*), and were distributed uniformly in their spectral tuning properties over the rat hearing range (Fig. 1*C*). Neurons exhibited reliable responses to 100 repeats of the stimuli (Fig. 4) as quantified by the signal-to-noise ratio, the standard deviation of the average firing rate divided by the average standard deviation of the firing rate across trials (in 10-ms bins), which averaged 2.8 for single units (*N* = 84) and 2.7 for all units (*N* = 211). A measure of response strength was used to characterize the neural behavior driven by each of the USVs (see methods; Fig. 5, *A* and *B*). Figure 4 and Fig. 5, *A* and *B*, provide examples that depict the response pattern and normalized response strength in two representative units. Of all units recorded, 27% were responsive (normalized response strength > 3) to at least one vocalization. The results were similar for the single units only: 24% were significantly responsive to at least one vocalization. *Unit 1* (Fig. 4, *B* and *C*; Fig. 5*A*) exhibited significant responses to *vocalizations 2*, *4*, *5*, *6*, *7*, and *8. Unit 2* (Fig. 4, *D* and *E*; Fig. 5*B*) exhibited significant responses to another subset of vocalizations (*3*, *4*, *8*). On average, neurons responsive to at least one USV were responsive to 1 or 2 of 8 (20%) USVs for multiunits and 2 or 3 of 8 (27%) USVs for single units (Fig. 5*C*). These data demonstrate that many A1 neurons significantly responded to the USVs, and that their responses were significant for only a subset of vocalizations.

To verify that the denoising procedure did not lead to activation of additional populations of neurons, we presented the original recordings of the USVs (containing background noise) alongside their denoised versions. The response strength of units was greater for vocalizations containing background noise than for noiseless vocalizations: of all units recorded in response to noisy vocalizations (*N* = 395 all units, *N* = 169 single units), 34% of all units (33% single units) were responsive to at least one noisy vocalization, suggesting that the background increased the responsiveness of neurons to the USVs. The response strength to vocalizations in the presence and absence of noise was significantly correlated over the population of neurons (*r* = 0.31, *P* < 1e-38, *N* = 210, all units; *r* = 0.39, *P* < 1e-24, *N* = 82 single units). These findings are consistent with previous reports on changes of A1 responses to vocalizations upon addition of background (Bar-Yosef and Nelken 2007). Since we were interested in analyzing the responses to USVs without the background, we used denoised vocalizations as stimuli for the remainder of the study.

### Differential Response of A1 Neurons to Eight Selected Ultrasonic Vocalizations is Not Correlated with Their Best Frequency

Tuning to a specific spectral band is an important response property of A1 neurons (Bizley et al. 2005; Brugge and Merzenich 1973; David et al. 2009; Ehret and Schreiner 1997; Recanzone et al. 1999; Schreiner 1992). We assayed whether the best frequency of A1 units, as determined from responses to tone pips of various frequencies, correlated with the response strength of neurons to USVs (*N* = 181 all units, *N* = 82 single units). The proportion of vocalizations to which the unit responded significantly (response-eliciting USV fraction, *R*_USV_) did not correlate significantly with the best frequency of the neurons [Fig. 5*D*; the correlation coefficient was not significant (*P* > 0.05) either for all units combined or for single units alone]. We further computed the overlap between the spectral response profile of the neuron and the power spectrum of each USV and compared it to *R*_USV_. The spectral response profile was determined as the response strength to tone pips presented at different frequencies. Across all vocalizations, for neurons that were significantly driven by at least one USV (*N* = 47 all units, *N* = 17 single units), *R*_USV_ was not significantly correlated with the degree of overlap of the USV power spectrum with the spectral response profile [Fig. 5*E*; the correlation coefficient was not significant (*P* > 0.05) either for all units combined or for single units alone]. The response selectivity index, *RS*, estimated as the maximum response strength to a USV divided by the mean response strength over all USVs, also did not exhibit significant correlation (data not shown) with either the best frequency [the correlation coefficient was not significant (*P* > 0.05) either for all units combined or for single units alone] or the spectral overlap between the USV power spectrum and the spectral response profile of the units [the correlation coefficient was not significant (*P* > 0.05) either for all units combined or for single units alone].

The eight USVs were similar in their power spectrum, with the dominant frequency within half an octave from each other (Fig. 4*A*). The differences among the USVs stemmed primarily from the changes in the temporal structure of the dominant frequency component. Thus differences in the discharge patterns of those units responsive to the USVs likely were driven by the modulation of the amplitude and frequency modulation in time, rather than the power spectrum.

### A1 Neurons Reliably Follow a Sequence of Vocalizations

**Fig. 6.**
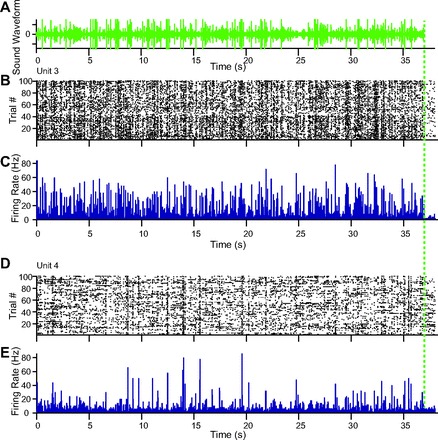
Responses to the long sequence of 350 concatenated USVs. *A*: stimulus waveform. *B*: raster plot of responses of *unit 3. C*: PSTH of responses of *unit 3. D*: raster plot of responses of *unit 4. E*: PSTH of responses of *unit 4*. Dashed vertical green line: end of stimulus.

To examine the responses of A1 neurons to temporally dynamic USVs, we composed a stimulus sequence consisting of 350 vocalizations (Fig. 3*A*, Fig. 6*A*, Fig. 8*A*). Figure 6 depicts the responses of two representative units to the vocalization sequence. Both of these units exhibited significant responses to the stimulus. The activity of many recorded units (all units *N* = 397, single units *N* = 172) was significantly modulated by the stimulus (signal-to-noise ratio > 2, 42% all units, 46% single units).

**Fig. 7.**
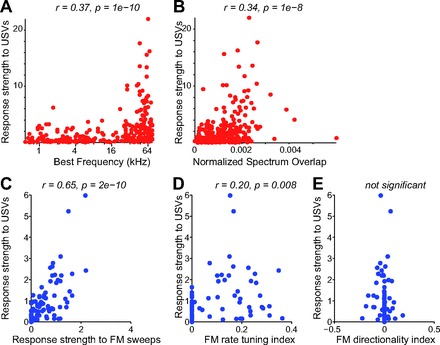
Correlation between the response strength to USVs and neuronal tuning properties. *A*: response strength to USVs vs. best frequency for all units. Each dot represents a single unit. *B*: response strength to USVs vs. the normalized overlap between its spectral response profile and the power spectrum of USVs. *C*: response strength to USVs vs. response strength to FM sweeps. *D*: response strength to USVs vs. response strength to FM rate tuning index. *E*: response strength to USVs vs. FM directionality index.

**Fig. 8.**
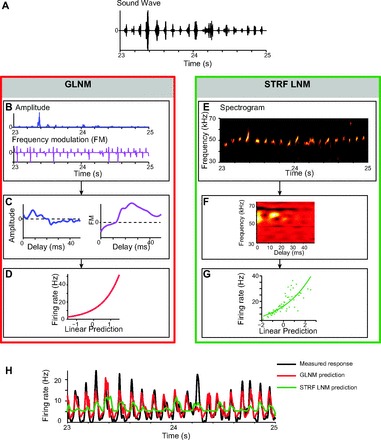
Predictive models of primary auditory cortex (A1) responses to vocalizations. *A*: sound wave of the stimulus. *B*: under the generalized linear-nonlinear model (GLNM), the stimulus is represented as a 2-dimensional vector, amplitude and frequency modulation, in time. *C*: 2 linear kernels are fitted to the responses of the unit to the first 200 vocalizations—1 for the amplitude (blue) and 1 for frequency modulation (purple). *D*: a single joint nonlinear response function is fitted as an exponential to the firing rate of response vs. the sum of the 2 linear predictions from the amplitude and frequency modulation vectors. *E*: under the spectro-temporal receptive field (STRF) linear-nonlinear model (LNM), the stimulus is represented by its spectrogram. *F*: STRF. *G*: nonlinear function is fitted as an instantaneous transfer function to the firing rate of responses vs. the linear prediction based on the STRF. *H*: prediction for firing rate of responses to the remaining 150 vocalizations. Red, GLNM model prediction; green, STRF LNM prediction; black, measured firing rate.

We compared the response strength of A1 neurons to the long USV sequence with the tuning properties for simpler stimuli, including tone pips and FM sweeps. Units exhibited significant correlation between their best frequency and response strength to USVs, such that units with best frequency in the ultrasonic range were most responsive to USVs (*r* = 0.37, *P* < 1e-10, *N* = 284 all units; *r* = 0.27, *P* < 1e-4, *N* = 125 single units; Fig. 7*A*). The normalized spectral overlap between the spectral response profile of the unit and the power spectrum of the USVs was also significantly correlated with response strength to the USVs (*r* = 0.34, *P* < 1e-8, all units; *r* = 0.28, *P* < 0.005, single units; Fig. 7*B*). Furthermore, units exhibited high correlation in their response strength to USVs and FM sweeps (*r* = 0.63, *P* < 1e-8, *N* = 74 units; *r* = 0.41, *P* < 0.05 single units, *N* = 32; Fig. 7*C*). The correlation between the FM rate tuning index and USV response strength was weaker across all units (*r* = 0.27, *P* < 0.05; Fig. 7*D*) and not significant for single units only (*P* > 0.05). This correlation is consistent with the observation that distinct USVs are restricted to a subset of FM rates. No significant correlation was observed between the FM directionality index and USV response strength (*P* > 0.05; Fig. 7*E*), as expected from the symmetrical distribution of USV spectral modulation in positive and negative direction (Fig. 3; Fig. 8*B*).

### A1 Responses Are Accurately Predicted by a Generalized Linear-Nonlinear Model

The computation that underlies generation of responses in A1 to vocalizations remains largely unknown. To characterize the computation by which A1 neurons produce responses to USVs, we next fitted two versions of linear-nonlinear model to the responses of each neuron during the first 200 vocalizations and then measured the prediction for the responses to the remaining 150 vocalizations (Fig. 8).

While many methods of fitting a linear-nonlinear model to neuronal responses exist, the advantage of using the generalized linear-nonlinear model (reduced GLNM) for these data is that the probability distribution of the input signal is not required to be Gaussian for the model to converge (Calabrese et al. 2011; Paninski et al. 2007). We developed a novel version of the GLNM, which was based on a low-dimensional representation of the stimulus, including the frequency modulation and the amplitude as functions of time. We compared the prediction accuracy of the GLNM to the predictions of a standard linear-nonlinear model (STRF LNM) based on the spectrogram of the stimulus. We used standard methods of reverse correlation to fit the spectro-temporal receptive field (STRF) (Escabi and Read 2003; Theunissen et al. 2001) and the instantaneous nonlinearity, which was not constrained in its shape (Geffen et al. 2009).

We first present the results of the fit for a representative neuron and then give the statistics over all recorded units. For the reduced GLNM, the stimulus was represented by two parameters: the amplitude and the frequency modulation (Fig. 8*B*). For the STRF LNM, the stimulus was represented as a spectrogram (Fig. 8*E*). Next, the linear filters were fitted under both models. For the sample unit in Fig. 8, both the amplitude and frequency modulation (Fig. 8*C*) filters follow an On-type time course (Geffen et al. 2007; Kuffler 1953), in which the peak of the filter is positive. The nonlinearity is fitted accurately by an exponential (Fig. 8*D*). In the STRF-based model, the STRF exhibits a delayed positive peak at about 60 kHz, but there is also a negative sideband present around 68 kHz (Fig. 8*F*). The prediction of the GLNM to novel USVs is remarkably accurate and more accurate than that of the STRF-based LNM (Fig. 8*H*).

**Fig. 9.**
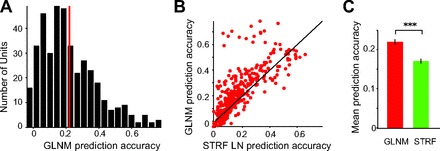
Over the population of A1 units, the GLNM accurately predicts neuronal responses. *A*: histogram of prediction accuracy for responses of all recorded units. Red bar, mean prediction accuracy. *B*: prediction accuracy of GLNM vs. STRF LNM. *C*: mean prediction accuracy of GLNM and spectrogram-based LNM. Error bars show SE. Significance level in a paired *t*-test: ***P < 0.001.

Over the population of neurons (all units *N* = 397, single units *N* = 172), the GLNM predicted responses to a novel stimulus sequence accurately (Fig. 9*A*; prediction accuracy up to 0.8) and significantly higher than the spectrogram-based model (Fig. 9, *B* and *C*; paired t-test, *P* < 1e-18 all units, *P* < 1e-8 single units). Across all recorded units, the mean coefficient of correlation between the GLNM and recorded firing rate was 0.22 (SE 0.01). This value was not significantly different from that for single units alone (0.19, SE 0.01); 28% of all neurons (21% of single units) fitted to the GLNM attained prediction accuracy higher than 0.3. The mean prediction accuracy of the spectrogram-based model was 0.17 for all units and 0.15 for single units (SE 0.01). Only 17% of all units (14% of single units) exhibited prediction accuracy higher than 0.3. Thus the responses to the vocalizations were accurately predicted by the reduced GLNM, and this prediction was more accurate than the prediction given by a spectrogram-based model.

### GLNM Prediction Depends on Tuning of A1 Units to Ultrasonic Frequencies

**Fig. 10.**
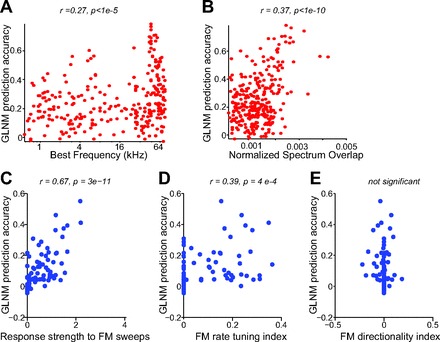
Correlation between model prediction accuracy and neuronal tuning properties. *A*: GLNM prediction accuracy vs. best frequency for each unit. *B*: GLNM prediction accuracy of each unit vs. the normalized overlap between its spectral response profile and the power spectrum of USVs. *C*: GLNM prediction accuracy vs. response strength to FM sweeps. *D*: GLNM prediction accuracy vs. response strength to FM rate tuning index. *E*: GLNM prediction accuracy vs. FM directionality index.

We next compared the prediction accuracy of the GLNM to the spectral and frequency modulation tuning properties of the units. There was a significant correlation (*r* = 0.27, *P* < 1e-5, *N* = 283; Fig. 10*A*) between the best frequency of the neuron and the model prediction accuracy across all units, but this correlation was not significant for single units alone (*P* > 0.05, *N* = 125). High prediction accuracy was exhibited by units whose best frequency was situated in the ultrasonic range of the vocalization: 54% of all units (28% of single units) whose best frequency was above 40 kHz exhibited prediction accuracy of 0.3 or higher; their mean prediction accuracy was 0.33 (0.24 for single units). Nevertheless, 23% of units (21% of single units), for which best frequency was below 40 Hz, exhibited a prediction accuracy of 0.3 or higher. Their mean prediction accuracy was 0.20 (0.21 for single units alone).

The model prediction accuracy was also significantly correlated with the overlap of the units' spectral receptive field and the power spectrum of the stimulus (Fig. 10*B*; *r* = 0.37, *P* < 1e-10) for all units but not for single units (*P* > 0.05). These findings demonstrate that best frequency plays a role in driving the responses of the units to the USVs, yet some units that are not tuned to the frequency range of vocalizations still exhibit predictable responses to USVs.

We also compared the model prediction accuracy to the FM sweep response properties of neurons. The model prediction accuracy was highly correlated with the FM sweep response strength (*r* = 0.66, *P* < 1e-7, *N* = 74; *r* = 0.45, *P* < 0.01, *N* = 32 single units; Fig. 10*C*) and significantly correlated with the FM rate tuning index (*r* = 0.37, *P* < 0.005, all units; Fig. 10*D*) for all units but not for single units alone. The correlation between the response strength to USVs and the FM rate tuning index (Fig. 7*D*) was consistent with the observed correlation between the FM rate tuning index and the model prediction accuracy (Fig. 10*D*), as the FM temporal filters were typically restricted to a specific subset of FM rate transitions (Fig. 8*C*). However, the FM directionality index did not correlate significantly with the model performance accuracy (*P* > 0.05; Fig. 10*E*).

### A1 Multiunits Respond More Strongly to Original than to Transformed USVs

One of the hallmarks of vocalization coding is the differential sensitivity of neurons to original and transformed vocalizations (Wang et al. 1995). Reversing a complex sound preserves all the first-order statistical features of the sound, yet the higher-level features are modified (second-order features are temporally reversed). For example, an upward frequency sweep, when reversed, becomes a downward frequency sweep; however, the spectral amplitude distribution and contrast remain unchanged. As coding of the stimuli takes into account more complex features, reversing the vocalization is expected to evoke a response different from the original signal.

**Fig. 11.**
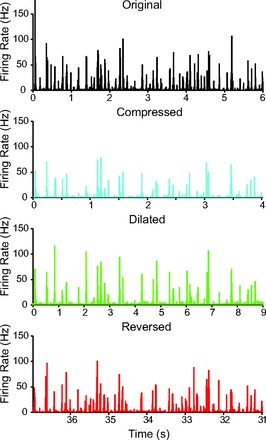
Time course of a representative unit to sequences of original and temporally transformed USVs. Firing rate of a representative unit in response to a segment of the USV sequence: black, responses to original USVs; cyan, compressed; green, dilated; red, reversed. To enable comparison of features of the stimulus the unit likely responded to, the time axes were transformed to compensate for the transformation imposed on the stimulus. For compressed condition, the time axis is expanded by ×1.5; for dilated condition, the time axis is compressed by ×0.67; for reversed condition, the time axis is reversed, and since the order of USVs was also reversed, the last segment is taken. These transformations, if applied to the sound waves, would have rendered them similar to each other.

To test whether A1 responds more strongly to the original than to transformed vocalizations, we presented a set of transformed vocalization sequences in which the vocalizations were *1*) accelerated (temporally compressed) by a factor of 1.5; *2*) slowed down (temporally dilated) by a factor of 0.67; *3*) reversed (see methods, Fig. 3). The responses of a representative unit to the four versions of the stimulus, transformed in time to compensate for the changes in the stimulus, are depicted in Fig. 11. Note that some of the peaks in the responses of the unit persist across the transformations of the vocalizations (indicating features in the stimulus that drive the unit regardless of the transformations), whereas other firing rate peaks do not repeat across transformation of vocalizations. A similar pattern of responses would be expected based on the GLNM of neuronal responses: Some features of USVs, when transformed and convolved with a linear filter (Fig. 8*B*), would be expected to result in a amplitude of activation similar to those resulting from original USVs, whereas other USV features would not.

**Fig. 12.**
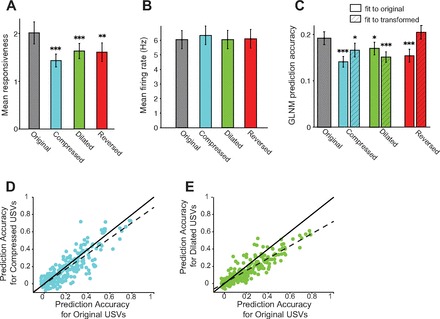
A1 exhibits preference for original over temporally transformed vocalizations. *A*: mean response strength of units to original, compressed (accelerated), dilated (slowed down), and reversed vocalizations. *B*: mean firing rate in response to original, compressed (accelerated), dilated (slowed down), and reversed vocalizations. *C*: mean prediction accuracy for responses to original, compressed (accelerated), dilated (slowed down), and reversed vocalizations. Solid bars, fitted on responses to original vocalizations; hatched bars, fitted on responses to transformed vocalizations. *A–C*: error bars show SE. Significance level in a repeated-measures ANOVA after Bonferroni multiple-comparison correction relative to original: ***P < 0.001, **P < 0.01, *P < 0.05. *D*: prediction accuracy for all units for responses to compressed vs. original USVs. Each dot denotes a unit. *E*: prediction accuracy for all units for responses to dilated vs. original USVs.

Across the population of cells, the response strength of units was lower for the transformed compared with the original USVs (*N* = 144, *P* < 0.05; Fig. 12*A*), while the mean firing rate did not change (repeated-measures ANOVA, *P* > 0.05; Fig. 12*B*). The difference in the response strength was not due simply to changes in the baseline firing rate or standard deviation, which did not exhibit significant changes across stimulus types (repeated-measures ANOVA, *P* > 0.05). For single units alone, the same trend was apparent (lower mean response strength for transformed compared with original vocalizations), but the differences in response strength were not statistically significant (*N* = 50, *P* > 0.05 after Bonferroni correction). The lack of change of the firing rate was also predicted by the GLNM fitted on the original vocalizations (repeated-measures ANOVA, *P* > 0.05). These results demonstrate that the original vocalizations elicited stronger response strength, although not a greater firing rate, compared with the transformed vocalizations in A1 neurons.

### GLNM Predicts A1 Neuronal Responses Less Accurately to Transformed Than to Original Vocalizations

We next examined the accuracy of the fit of the GLNM for responses to transformed USVs when fit on the original USVs. We first fitted the predictions of the GLNM on the first 200 original USVs and used these fits to generate a prediction for USV responses to the remaining 150 transformed vocalizations. The model predictions were less accurate for the reversed, compressed, and dilated vocalizations (*N* = 144, all units, *P* < 0.05, repeated-measures ANOVA, Bonferroni multiple-comparison corrected, same trend but comparison not significant for single units alone; Fig. 12*C*).

However, the reduced model prediction accuracy change could potentially be due simply to undersampling of the stimulus space during the model fit. To verify that the prediction accuracy is indeed decreased because of a change in the response parameters, we fitted the model on the first 200 vocalizations of each of the transformed stimuli and tested it on the remaining 150 under each condition. After this change in analysis, the decrease in prediction accuracy was still significant for the dilated and compressed vocalizations (Fig. 12, *C–E*; *P* < 0.05 after Bonferroni multiple-comparison correction, all units combined, but not significant for single units alone). The differences of processing of stimuli following temporal dilation and compression point to a differential processing mechanism for original and temporally transformed vocalizations. However, for reversed vocalizations, the prediction accuracy was consistent with that of the original stimuli (difference was not significant at *P* < 0.05 after Bonferroni correction) when the GLNM was fitted on the reverse vocalizations. This is consistent with the explanation that the original stimuli undersampled the statistical space of reverse stimuli. Combined, these results indicate that the integration of the amplitude and dominant frequency modulation of the USVs best predicts the responses of A1 neurons to original vocalizations, but the computations underlying this integration favor the natural statistics of the vocalizations.

## DISCUSSION

Rats communicate with USVs, yet despite their behavioral prevalence, little is known about how USVs are encoded in the auditory system. Here, we characterized the responses of neurons in the awake rat A1 to distinct USVs. A1 neurons exhibited significant responses to a subset of USVs. We found that the response strength of neurons in A1 to USVs was correlated with their best frequency, FM sweep response strength, and FM rate tuning index. We constructed a reduced-parameter predictive model, which relied on temporal integration of dominant frequency modulation and amplitude of the signal. This model accurately predicted the responses of A1 neurons to previously unheard vocalizations. These results contribute a significant advance over previous work on encoding of conspecific vocalizations in A1 by demonstrating a simple, yet precise computation that underlies their encoding. We also present evidence for preference in A1 responses to the temporal statistics of the original USVs: A1 responses had higher response strength and the prediction of the model was more accurate for original compared with temporally transformed vocalizations. Our findings suggest that the neuronal circuits of processing ultrasonic sounds are tuned to the ethologically relevant stimulus statistics.

### Preference of A1 Responses for Temporal Structure of Original USVs

The hierarchical theory of cortical processing (Felleman and Van Essen 1991; Mineault et al. 2012; Zeki and Shipp 1988) posits that neural networks in more central auditory cortical areas encode progressively more complex features of the stimulus, increasing their preference for complex, specific acoustic objects, such as vocalizations (Hackett 2011; Kaas and Hackett 1998; Leaver and Rauschecker 2010; Recanzone 2008; Rouiller et al. 1991; Ter-Mikaelian et al. 2007). Indeed, for some species, including the nonhuman primate, it has been shown that neurons in A1 respond more strongly to the original rather than temporally transformed (compressed or dilated) vocalizations (Wang 2000).

The auditory system also exhibits tolerance to temporal transformations of acoustic stimuli. In humans, speech comprehension does not degrade perceptual accuracy for as much as a twofold compression (Beasley et al. 1980; Foulke and Sticht 1969). At the neurophysiological level, it has been shown that cells in A1 exhibit envelope following of the stimulus (Ahissar et al. 2001; Gehr et al. 2000; Gourevitch and Eggermont 2007; Mukamel et al. 2011; Nourski et al. 2009), thereby preserving their firing rate to original and temporally transformed stimuli.

Our results argue for a preference of neuronal responses in A1 to original over temporally compressed or dilated vocalizations. In evaluating the response strength of A1 neurons to different vocalizations, we found that the response strength, characterized by the relative variation in the firing rate compared with the baseline, was greater for original than for temporally dilated or compressed USVs (Fig. 12*A*). On the other hand, the absolute mean firing rate during stimulus presentation did not vary significantly across the conditions (Fig. 12*B*), which may be due to a mechanism that serves to maintain a constant response rate in neurons under different statistics of the stimulus, such as divisive normalization.

Our predictive model fits revealed a further statistically significant dependence of responses on the temporal structure of the stimulus. When a long vocalization sequence was transformed in temporal statistics, the accuracy of the predictive model of neuronal responses decreased significantly (Fig. 12*C*). Thus the response properties of neurons depended on the statistical makeup of the stimulus features. This dependence was not likely to be due to a simple temporal dilation or compression of the temporal kernel, as refitting the model on the transformed vocalizations preserved the decrease in the accuracy of the model's performance. The accurate fit of the predictive model for responses to original USVs demonstrates that the responses in A1 neurons reflect a computation, based on integration of the time course of frequency modulation and the amplitude of the dominant spectral component. The decrease in the accuracy of the prediction of the model for responses to transformed vocalizations suggests that the parameters of this computation are tuned to the statistics of the original, rather than transformed, USVs. Our model identifies a simple mechanism that likely leads to A1 responses, and allows for comparison of responses across stimulus types with varying statistical structure. Future studies are needed to determine whether the preference for original USVs by A1 neurons results in increased coding efficiency within this ethologically relevant statistical regime, as predicted by the efficient coding hypothesis (Barlow 1961).

### Similar Computation Underlying Responses to Reversed Vocalizations

Previously, it has been found that A1 neurons exhibit a stronger response to the original vocalizations than to their reversed versions in nonhuman primates (Averbeck and Romanski 2006; Romanski and Averbeck 2009; Wang and Kadia 2001; Wang et al. 1995). This preference for original vocalizations, which may partially be explained by differences in spectro-temporal structure of the original and reversed vocalizations, has been taken as a hallmark of vocalization specificity. However, other studies did not find a similar preference for the original over reversed vocalizations (Huetz et al. 2009; Schnupp et al. 2006). To test whether the high accuracy in response prediction of the neurons was specific to the original vocalizations, we presented a stimulus sequence consisting of reversed vocalizations (Fig. 3*D*). Like temporally transformed vocalizations, we found that response strength of the neurons, but not the mean firing rate, was decreased during the presentation of reversed USVs compared with the original (Fig. 12, *A* and *B*). Unlike temporally transformed USVs, the reduced response strength was not accompanied by reduced model prediction accuracy, as would be predicted were the neurons to respond to the reversed USVs via a differential mechanism (Fig. 12*C*). These results suggest that the original and reversed vocalization stimuli, which overlap in their temporal and frequency modulation spectrum, covering the same range (Fig. 3), appear to activate A1 via a similar computational mechanism.

### Response Strength of A1 Neurons to USVs

A previous study in the mouse A1 found that neurons located in the sonic range of the tonotopic axis were responsive to ultrasonic tones, even if their best frequency was found in the sonic range (Linden et al. 2003). We also found that many neurons whose best frequency was outside the ultrasonic range responded significantly to USVs. However, the responses of neurons whose best frequency overlapped with the power spectrum of the USVs were more accurately predicted by the GLNM—suggesting that their responses are likely controlled by a reduced set of precise computations, as evidenced by our predictive model.

Our results extend the previous characterization of responses to a different type of USVs in the mouse (Holmstrom et al. 2010; Lin and Liu 2010; Liu and Schreiner 2007; Liu et al. 2006). A subset of A1 neurons in the mouse exhibit precise temporal following and short response onset latency in response to ultrasonic infant pips (Lin and Liu 2010). Our GLNM may provide for the computation that produces the reduced onset latency: reduced response onset latency may be predicted by our GLNM if both frequency modulation and amplitude kernels were to exhibit a short time to peak. The correlation of the identity of the neurons with their response strength and predictability to USVs remains to be experimentally tested.

### Neuronal Correlates of the GLNM

The GLNM was partially based on integration of frequency modulation and amplitude of the dominant spectral channel over time. Not surprisingly, the response strength to the USVs in A1 was correlated strongly with response strength to FM sweeps. Therefore, the mechanisms that underlie the computation identified in the GLNM may share neuronal circuits with those giving rise to FM responses. FM responses in A1 have been proposed to have both subcortical as well as cortical origins (Atencio et al. 2007; Nelken and Versnel 2000; Phillips et al. 1985; Shamma et al. 1993; Trujillo et al. 2011; Zhang et al. 2003). A recent study demonstrated that direction tuning of A1 responses may be explained by integration of the incoming signals from the ascending afferent auditory pathway, which are already directionally tuned (Kuo and Wu 2012). These signals may originate as early as the inferior colliculus, a brain area peripheral to A1 where neurons are tuned to specific direction of frequency modulation of the incoming sound (Clopton and Winfield 1974; Hage and Ehret 2003; Rees and Moller 1983; Suga 1968). However, additional mechanisms that generate frequency modulation tuning, including sideband facilitation and inhibition (Razak and Fuzessery 2006, 2008), may also be at play in driving the responses to USVs within A1. Sideband facilitation and inhibition may also be behind the responses to integration of amplitude modulations of those neurons that do not exhibit tuning to ultrasonic tone pips. These mechanisms, carried out by intracortical connections, have previously been shown to give rise or modulate responses in A1 neurons to signals outside the center of their frequency response area (summarized in Schreiner et al. 2011; Sutter 2005) and may drive responses of A1 neurons to amplitude modulations in USVs.

The low-parameter description of the signal allowed us to represent the sound with 1-ms precision and preserve high-frequency resolution in representing the frequency modulation, while implementing the computationally intensive maximum likelihood optimization technique. To arrive at this representation, the signal was transformed through a nonlinear operation (preceding the fit of the GLNM): extraction of the maximum instantaneous frequency of the signal. It is plausible that the rat auditory system implements a similar operation in isolating the vocalization from the background noise. The neuronal correlate of this operation would involve a potential winner-take-all circuit, implemented through, for example, lateral inhibition across direction-tuned neurons (Xie et al. 2002).

These mechanisms may generalize to more complex vocalizations that contain multiple spectral components such as harmonics, and to environmental sounds, which contain broadband components (Geffen et al. 2011). Our model may be extended to include an intracortical lateral inhibitory circuit that would detect the maximally activated direction-tuned channels. This mechanism would allow for encoding of multiple dominant spectral components, separated across several channels. Signal in each channel would then undergo a processing cascade, described by a GLNM, based on frequency modulation and amplitude of each distinct channel. Further studies of processing of harmonic vocalizations are needed to determine whether the computation proposed in this paper generalizes to harmonic and environmental sounds.

In this work, we explored the computation that underlies the responses of neurons in A1 to USVs. We found that the computation relies on integration of the time course of frequency modulation and amplitude of the dominant spectral component of the USVs. The use of USVs as stimuli enabled us to develop a simple, yet powerful model of A1 responses. A similar reduction of the parameters of the stimulus may prove powerful in predicting and understanding the mechanisms of A1 responses for other types of stimuli, including speech and music. The predictability and precision of responses of individual neurons further corroborate the role of A1 as a brain area important for extracting behaviorally meaningful acoustic information.

## GRANTS

M. N. Geffen is the recipient of the Burroughs Wellcome Career at the Scientific Interface award. The work was partially supported by the Pennsylvania Lions Hearing Research grant, the Klingenstein Award for the Neurosciences, and the University of Pennsylvania Center for Collaborative Neuroscience Pilot Grant to M. N. Geffen as well as the National Institutes of Health Computational Neuroscience Training Grant and the National Science Foundation Integrative Graduate Education and Research Traineeship (IGERT) Training Grant in Complex Scenes and Perception to I. M. Carruthers.

## DISCLOSURES

No conflicts of interest, financial or otherwise, are declared by the author(s).

## AUTHOR CONTRIBUTIONS

I.M.C. and M.N.G. conception and design of research; I.M.C., R.G.N., and M.N.G. performed experiments; I.M.C., R.G.N., and M.N.G. analyzed data; I.M.C., R.G.N., and M.N.G. interpreted results of experiments; I.M.C., R.G.N., and M.N.G. prepared figures; I.M.C. and M.N.G. drafted manuscript; I.M.C. and M.N.G. edited and revised manuscript; I.M.C., R.G.N., and M.N.G. approved final version of manuscript.
